# *Helicobacter pylori* infection and hypochlorhydria in Zambian adults and children: A secondary data analysis

**DOI:** 10.1371/journal.pone.0256487

**Published:** 2021-08-27

**Authors:** Phoebe Hodges, Paul Kelly, Violet Kayamba

**Affiliations:** 1 Tropical Gastroenterology & Nutrition group, University of Zambia School of Medicine Department of Internal Medicine, Lusaka, Zambia; 2 Blizard Institute, Barts & The London School of Medicine and Dentistry, Queen Mary University of London, London, United Kingdom; Osaka City University Graduate School of Medicine, JAPAN

## Abstract

**Background:**

Hypochlorhydria (gastric pH >4) increases susceptibility to diarrhoea, iron deficiency, and gastric cancer. We sought to clarify the prevalence of this condition and its predisposing factors in Zambia by pooling data from previous studies conducted in hospital and community settings.

**Methods:**

Gastric pH was measured in participants from five separate studies by collecting gastric aspirate from fasted adults and children under 3 years of age undergoing gastroscopy. Gastric pH was correlated with serological testing for Human Immunodeficiency Virus (HIV) and *Helicobacter pylori* (*H*. *pylori*) infections.

**Results:**

We studied 597 individuals (487 adults and 110 children). Hypochlorhydria was present in 53% of adults and 31% of children. HIV infection was detected in 41% of adults and 11% of children. *H*. *pylori* serology was available for 366 individuals: 93% of adults and 6% of children were seropositive. In univariate analysis, hypochlorhydria was significantly associated with HIV seropositivity (OR 1.7; 95% CI 1.2–2.4; p = 0.004) and *H*. *pylori* antibody seropositivity (OR 4.9; 95% CI 2.8–8.6; p<0.0001), and with advancing age in HIV negative individuals (p = 0.0001). In multivariable analysis, only *H*. *pylori* was associated with hypochlorhydria (OR 4.0; 95% CI 2.2–7.2; p<0.0001) while excluding possible exposure to proton pump inhibitors.

**Conclusions:**

Hypochlorhydria is common in our population, with *H*. *pylori* being the dominant factor. Only young HIV seronegative individuals had a low prevalence of hypochlorhydria. This may have implications for the risk of other health conditions including gastric cancer.

## Introduction

Hydrochloric acid is produced by gastric parietal cells maintaining an acid environment in the stomach. This protects against ingested pathogens, facilitates initiation of protein digestion, and mediates absorption of iron, calcium, vitamin B12, and certain medications. Acetylcholine, histamine, and gastrin stimulate gastric acid production while prostaglandin E2 and somatostatin inhibit it. Hypochlorhydria is the failure of normal gastric acid production, either as a loss of normal physiological function or due to gastric atrophy [1].

Globally, the prevalence of hypochlorhydria (gastric pH greater than 4) is estimated to be less than 5% in young adults and increases to as much as 12% in the elderly [2]. Several factors cause hypochlorhydria, including pernicious anaemia, (autoimmune condition against parietal cells), *Helicobacter pylori (H*. *pylori)* infection, endocrine tumours secreting vasoactive intestinal peptide, and some iatrogenic causes including acid-suppressing medication, gastric bypass surgery, and radiation [2]. There is no known gender predilection for hypochlorhydria [3] but some investigators found differences in responsiveness to hypochlorhydria between males and females, with a suggestion that it could contribute to the differential prevalence of dyspeptic symptoms between the two sexes [4]. There have been relatively few studies reporting hypochlorhydria in children and the effect of *H*. *pylori* infection on acid secretion in children is still poorly defined [5].

There is evidence linking persistent hypochlorhydria to gastric carcinogenesis [6] mostly as a consequence of atrophy. An example of such experimental evidence was demonstrated by studying one-year-old Kcne2(-/-) mice in a pathogen-free environment [7]. KCNQ1 alpha subunit and the KCNE2 beta subunit provide a K^+^ efflux current to facilitate gastric acid secretion by the apical H^+^K^+^ATPase in parietal cells. These mice exhibited increased gastric mass with a severe gastric pre-neoplastic phenotype. In addition, some mice exhibited evidence of neoplastic invasion [7]. In humans, a study of close relatives of gastric cancer patients found that the prevalence of hypochlorhydria was 27%, which was much higher than that of healthy controls (3%) [8]. Iron deficiency is another well described consequence of hypochlorhydria [9], contributing to the global burden of anaemia [10].

The prevalence of hypochlorhydria in Africa is unclear, but previous studies have suggested high figures associated with Human Immunodeficiency Virus (HIV) [11, 12]. A study from Malawi did not find this link but instead reported an association with *H*. *pylori* infection [13]. This is an important association as WHO classifies *H*. *pylori* infection as a Class I (definite) gastric carcinogen and its prevalence is known to be higher in developing countries; indeed a systematic review and meta-analysis found a pooled prevalence of 70% across the African continent [14]. A study conducted in Chile using faecal antigen testing found that acquisition of *H*. *pylori* infection may occur as early as the first month of life [15].

In this study, we performed a secondary analysis of published and unpublished data on hypochlorhydria from a single centre in Lusaka, Zambia.

## Methods

We included data from five studies, all of them carried out in the endoscopy unit of the University Teaching Hospital, Lusaka. In all cases, oesophagogastroduodenoscopy (OGD) was performed using a Pentax 2990i video gastroscope or Pentax 2490i paediatric gastroscope, under sedation. Sedation in children was provided by an anaesthetist [16]. During the OGD procedure, gastric juice was aspirated after flushing the working channel, and the pH (to the nearest 0.5) was measured using pH paper test strips (Sigma Chemical Company St Louis, USA). Interviewer-administered questionnaires were used to collect basic patient information. Serum was tested for HIV antibodies using Uni-Gold^™^ rapid diagnostic kits (Trinity Biotech, Wicklow, Ireland). IgG antibodies against *H*. *pylori* were quantitatively measured in U/mL with Enzyme-Linked Immunosorbent Assay (ELISA), by Biohit Helsinki, Finland.

Each of the studies whose data were included evaluated gastric pH. This approach enabled us to improve the precision of estimates of effect, and facilitate analysis of a larger data set improving the confidence that it is a true representation of the population under study.

For all study participants, informed and written consent was obtained for study participation. For children, written consent was obtained from their parents or legal guardians. Consent was obtained in accordance with the requirements of the University of Zambia Biomedical Research Ethics Committee (UNZABREC).

### Study 1: Dyspeptic individuals presenting for oesophagogastroduodenoscopy

Patients above 18 years old (n = 247) referred for dyspepsia who had had a normal OGD were enrolled [17]. These participants were questioned about history of proton pump inhibitor (PPI) use at the time of enrolment. Samples in this study were collected between 2016 and 2018. The study protocol was approved by UNZABREC, reference number 000-03-16) and the National Health Research Authority (NHRA).

### Study 2: Asymptomatic HIV infected patients presenting for regular clinic follow-up

Asymptomatic patients presenting to the HIV clinic for scheduled visits were enrolled in this study [11]. Individuals above the age of 18 years who had given written consent were included and invited to come for OGD (n = 84). Samples in this study were collected from 2014 to 2015. UNZABREC (ref no. 004-04-14) and NHRA approved this study. These participants were not taking PPI medication.

### Study 3: Community volunteers

This was a study of adults (2003–2006) in Misisi, an impoverished high-density residential area in Lusaka [18]. Adults were recruited into a randomised controlled trial of multiple micronutrient supplementation, and endoscopy was performed yearly. The trial was registered as ISRCTN31173864. For this study, the first endoscopy performed for that individual was selected. Gastric pH did not differ by treatment allocation (median pH 2.25 (IQR 1–5) in the micronutrient supplementation group, and 3.0 (IQR 1–5.5) in the placebo group; P = 0.31), so these data (n = 156) are considered together. These participants were not taking PPIs. The study was approved by UNZABREC (ref 001-06-02).

### Study 4: Children with severe acute malnutrition

This study included children with severe acute malnutrition (SAM) (n = 25) in the malnutrition ward of the University Teaching Hospital, Lusaka [19]. Only children with complicated SAM are admitted to this ward, and all these children were being investigated for persistent diarrhoea associated with SAM. These participants were not taking PPIs. The study was approved by UNZABREC and NHRA (ref 006-01-13) and was conducted between 2013 and 2017.

### Study 5: Children with stunting or wasting

Gastric pH was measured as part of a protocol for investigating causes of failure to thrive in children with stunting (n = 85) in Misisi, as previously reported [16, 20]. In brief, children were recruited if they had evidence of stunting (length-for-age z score less than -2) or underweight (weight-for-age z score less than -2). They were provided with daily corn-soya blend, egg, and micronutrient supplements and investigated if there was no response in linear growth, so all these children were undergoing endoscopy on the basis that they were stunted and were non-responsive. These participants were not taking PPIs. The study was approved by UNZABREC (ref 006-02-16) and the NHRA and it was conducted between 2016 and 2019.

### Data analysis

Anthropometric measurements were reported as median (IQR) because these data were not normally distributed tested using the Shapiro–Wilk test. Mann Whitney U and Kruskal Wallis H tests were used for the comparison of non-parametric variables between groups. Spearman rank-order correlation coefficient was used for correlation between non-parametric variables. Chi-square was used for testing the relationship between categorical variables. Univariate and multivaraible logistic regression was used to adjust for possible confounders. In all cases, p-values less than 0.05 were considered statistically significant. Data were analyzed in SPSS version 26 and Stata 15.1.

## Results

### Basic demographic data for study participants

We obtained gastric pH data for 597 individuals: 487 adults and 110 children (Table 1). Of these, 247 (41%) were from study 1, 84 (14%) study 2, 156 (26%) study 3, 25 (4%) study 4 and 85 (14%) study 5. From study 1, PPI use was reported in 163 of the participants. The median age among adult patients was 43 years (IQR 34–54 years) while that among children was 19 months (IQR 14–22 months). The proportion of females was 281 (58%) among adults and 59 (54%) among children.

**Table 1:** Patient characteristics of adults and children included in the study.

Variable		Number		
Adults (n = 487)				
Sex		Study 1 (n = 247)	Study 2 (n = 84)	Study 3 (n = 156)
	Males	115 (47%)	29 (35%)	62 (40%)
	Females	132 (53%)	55 (65%)	94 (60%)
Age in years	<30	10 (4%)	8 (10%)	55 (35%)
	30–44	88 (36%)	42 (50%)	60 (39%)
	45–59	84 (34%)	29 (34%)	31 (20%)
	60 and above	65 (26%)	5 (6%)	10 (6%)
Body mass index, kg/m^2^; median (IQR)		25.0 (21.5, 28.9)	22.4 (20.6, 27.2)	21.9 (19.9, 25.6)
Gastric pH	< or = 4	94 (38%)	36 (43%)	101 (65%)
	>4	153 (62%)	48 (57%)	55 (35%)
HIV	Positive	42 (17%)	84 (100%)	63 (40%)
	Negative	180 (73%)	0 (0%)	88 (56%)
	Missing	25 (10%)	0 (0%)	5 (4%)
*Helicobacter pylori* infection	Positive	198 (80%)	67 (80%)	-
	Negative	4 (2%)	17 (20%)	-
	Missing	45 (18%)	0 (0%)	-
Recent PPI use		163	0	0
Children (n = 110)				
Sex		Study 4 (n = 25)	Study 5 (n = 85)	
	Males	13 (52%)	38 (45%)	
	Females	12 (48%)	47 (55%)	
Age in months	<1	5 (20%)	9 (11%)	
	1.0–1.4	7 (28%)	22 (26%)	
	1.5–1.9	13 (52%)	42 (49%)	
	2.0 and above	0 (0%)	12 (14%)	
Weight for length z score		-2.94 (-4.27, -2.05)	-0.64 (-1.27, -0.22)	
Weight for age z score		-3.55 (-4.95, -2.73)	-2.34 (-2.75, -1.86)	
Length for age z score		-2.48 (-3.88, -1.77)	-3.34 (-3.96, -2.79)	
Gastric pH	< or = 4	12 (48%)	64 (75%)	
	>4	13 (52%)	21 (25%)	
HIV	Positive	11 (44%)	1 (1%)	
	Negative	14 (56%)	84 (99%)	
*Helicobacter pylori* infection	Positive	-	5 (6%)	
	Negative	-	75 (88%)	
	Missing	-	5 (6%)	

*None of the children had a history of PPI use.

### Hypochlorhydria and gastric pH

Hypochlorhydria (pH>4) was present in 256 (53%) of the adults and 34 (31%) of the children (OR for childhood 0.4; 95% CI 0.3–0.6, p < 0.001). The median gastric pH among adults was 5.0 (IQR 1.5–6.0) while among children it was 2.5 (IQR 1.5–4.5) (p < 0.001 using Mann Whitney U test).

Categorising by study; the prevalence of hypochlorhydria was 62% in study 1, 57% in study 2, 35% in study 3, 52% in study 4, and 25% in study 5, (Table 1).

The prevalence of hypochlorhydria was 201/331 (61%) among adults enrolled from the community and 55/156 (35%) among adults recruited from the hospital. The difference was statistically significant (OR 2.8; 95% CI 1.9–4.3, p<0.001). Similarly, the prevalence of hypochlorhydria was higher among children recruited from the hospital than those from the community (OR 3.3; 95% CI 1.2–9.2, p = 0.02).

Concerning the impact of age, we remarked that this factor was associated with gastric pH in adults (Spearman ρ 0.16 (95% CI 0.1–0.3); p = 0.001) but not children (Spearman ρ -0.07 (95% CI -0.2- (-0.09)); p = 0.35).

**Fig 1:**
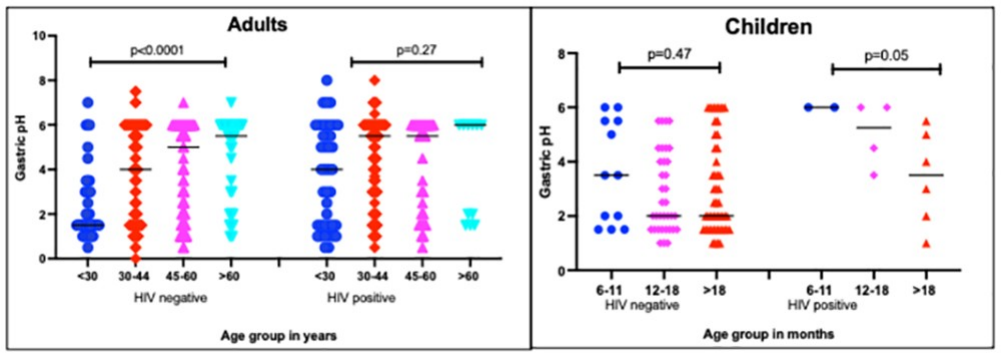
Gastric pH categorized by age group in HIV positive and negative adults and children. *Black horizonatal lines across each set of points represent the median ** The p-values reported were generated using the Kruskal-Wallis test evaluating association between gastric pH and the respective age groups.

When categorized by HIV infection, the association between age and gastric pH was only evident in the group of adults with HIV-negative status (p<0.0001) Fig 1. Hypochlorhydria was significantly higher with advancing age ranges in HIV-negative adults, Table 2. In children, there was a significant association between wasting and hypochlorhydria (OR 4.1; 95% CI 1.5–11.7, p = 0.002).

**Table 2:** Association between age and gastric pH in individuals with or without HIV infection.

Age group	Hypochlorhydria; n (%)	p-value
HIV negative		
<30 years	6 (15)	0.0001
30–44 years	43 (49)	
45–60 years	45 (53)	
Above 60 years	39 (68)	
HIV positive		
<30 years	18 (44)	0.19
30–44 years	52 (60)	
45–60 years	31 (61)	
Above 60 years	6 (55)	
HIV negative		
6–11 months	5 (42)	0.71
12–18 months	8 (23)	
>18 months	14 (27)	
HIV positive		
6–11 months	2 (100)	0.08
12–18 months	3 (75)	
>18 months	2 (33)	

*Tests for significance evaluated using the Kruskal-Wallis test.

### Association of hypochlorhydria with *Helicobacter pylori* (*H*. *pylori*) and HIV infections

The median serum concentration of *H*. *pylori* antibodies was available only in studies 1, 2, and 5. The levels were 116 U/mL (IQR 76–148) among adults and 8U/mL (IRQ 4–16) among children, (p < 0.001 by Mann-Whitney U test). There was a significant association between *H*. *pylori* positivity (antibody >30 U/mL) and hypochlorhydria across all age groups (OR 4.9; 95% CI 2.8–8.6; p<0.0001).

There was a significant association between HIV infection and hypochlorhydria (OR 1.7; 95% CI 1.2–2.4; p = 0.004). This association was only present in children when participants were separated into age groups (Table 3). Overall, 104 study participants had both *H*. *pylori* and HIV infections, 156 had *H*. *pylori* without HIV, 18 had HIV without *H*. *pylori* and 78 had neither infection. There was no increase in odds of hypochlorhydria in individuals with HIV and *H*. *pylori* co-infection compared to *H*. *pylori* alone (OR 1.3; 95% CI 0.8–2.3; p = 0.36).

**Table 3:** Logistic regression analysis by infection with *H*. *pylori* or HIV.

**All participants**	Hypochlorhydria (pH>4)			Univariate		Multivariable	
		Yes	No	OR (95%CI)	p value	OR (95%CI)	p value
*H*. *pylori* infection	Yes	171	99	4.9 (2.8–8.6)	<0.001	4.6 (2.7–7.8)	<0.001
	No	25	71				
HIV infection	Yes	114	87	1.7 (1.2–2.4)	0.004	1.3 (0.8–2.1)	0.26
	No	160	206				
**Adults**	Hypochlorhydria (pH>4)			Univariate		Multivariable	
		Yes	No	OR	p value	OR	p value
*H*. *pylori* infection	Yes	170	95	3.6 (1.3–10.8)	0.006	3.8 (1.5–10.1)	0.006
	No	7	14				
HIV infection	Yes	107	133	1.3 (0.9–0.2)	0.15	1.2 (0.7–2.0)	0.46
	No	82	135				
**Children**	Hypochlorhydria (pH>4)			Univariate		Multivariable	
		Yes	No	OR	p value	OR	p value
*H*. *pylori* infection	Yes	1	4	0.8 (0.01–8.7)	1.0	0.8 (0.08–7.4)	0.83
	No	18	57				
HIV infection	Yes	5	7	3.7 (0.9–15.8)	0.04	-	-
	No	27	71				

*Variables included in the multivariable analysis were HIV and *H*. *pylori* infections only.

When adults with recent exposure to PPIs were excluded from the analysis, the percentage with hypochlorhydria was 47%. Median gastric pH among adults with no history of PPI exposure was 4.0 (IQR 1.5–6.0). The significant association between hypochlorhydria and age greater than 30 years in HIV negative individuals remained when individuals with recent PPI exposure were excluded from analysis (p<0.001), as did the positive correlation between gastric pH and age in HIV negative adults (Spearman ρ = 0.25 (95% CI 0.11–0.38); p < 0.001). The associations observed with *H*. *pylori* infection and HIV were also still present (OR 4.7; 95% CI 2.6–8.4; p < 0.001 for *H*. *pylori*, OR 2.4; 95% CI 1.6–3.6; p < 0.001 for HIV).

In multivariable logistic regression analysis excluding individuals with a history of PPI use and adjusting for age, sex and HIV status, only *H*. *pylori* was noted to be associated with hypochlorhydria (OR 4.0; 95% CI 2.2–7.2; p<0.0001). According to adults and children’s groups, multivariable analysis models showed a positive association between hypochlorhydria and *H*. *pylori* in the group of adults (OR 3.4; 95% CI 1.1–10.3; p = 0.03) but not children (OR 0.8; 95% CI 0.1–7.9, p = 0.84).

## Discussion

There are well-established health implications of hypochlorhydria with evidence drawn from both animal and human studies. We pooled data from endoscopic studies that previously evaluated gastric pH among Zambian adults and children. We found that the occurrence of hypochlorhydria was associated with *H*. *pylori* infection in both adults and children. We also found an association between hypochlorhydria and age greater than 30 years in HIV negative individuals.

Our data, despite being from different studies, are comparable as we collected the samples using a similar protocol. The data from study groups 1 and 3 represent unselected adults, but they may not be fully representative of the population: participants in study 1 may have had other causes for symptoms even though OGD was normal, and study 3 was from an area with very low socioeconomic status. We previously reported a high prevalence of hypochlorhydria in Zambian adults, which was associated with HIV infection [11, 12]. Our current pooled analysis has further demonstrated that the difference in gastric pH between HIV positive and negative individuals is most pronounced in young adults below the age of 30 years and not in the older age groups. Our data are suggesting that HIV significantly reduces the age at which hypochlorhydria starts to develop. In addition, we have published evidence that HIV-associated hypochlorhydria is not altered by anti-retroviral therapy (ART). In our previous study, the proportion of hypochlorhydria in ART naïve patients was similar to those with ART-induced viral load suppression [11]. However, it is not clear if the high occurrence of hypochlorhydria among Zambians has had any impact on response to ART regimes that include weakly basic drugs such as atazanavir, nelfinavir, raltegravir, or delavirdine. These later have low solubility at high pH hence hypochlorhydria may lead to less efficient absorption across the gastric mucosa and lower bioavailability [21, 22]. If this is the case it could have significant implications particularly in this population with high HIV seroprevalence. There are also implications for the impact on the bioavailability of other widely-used weakly basic drugs including some antibiotics and antifungals (ceftriaxone, ketoconazole, itraconazole) as well as drugs used in cardiovascular conditions including digoxin and dabigatran [22].

Hypochlorhydria may also result in increased absorption of other drugs, with one study of PPI-induced hypochlorhydria demonstrating increased cumulative fractions of theophylline absorbed from a sustained-release preparation during 3.5 hours [23]. This has clear implications for patients with hypochlorhydria (even where this is not PPI-related) who are taking these drugs long-term as they may be at increased risk of toxicity.

Our data shows a positive association between hypochlorhydria and *H*. *pylori* infection which consolidate with other studies [24, 25]. *H*. *pylori* is known to have a suppressive effect on gastric acid secretion. Moreover, it is reported that the treatment against this pathogen leads to an improvement in the production of stomach acid [26]. Decreased production of stomach acid is caused by many factors, including the suppression of the H^+^K^+^ATPase pump, the stimulation of inflammatory cytokines including interleukin-1B, atrophic gastritis that destroys acid-secreting parietal cells [27–29], and the production of urease by *H*. *pylori*, an enzyme that hydrolyses urea in the stomach to produce ammonia and then ammonium, to neutralize the gastric acidity allowing bacterium surviving [30].

We found no association between coinfection with HIV and *H*. *pylori* and increased risk of hypochlorhydria as was previously demonstrated in a study of Malawian adults [13]. However, analysis of gastric biopsies in the Malawian cohort demonstrated that co-infection was associated with pangastritis (contrary to *H*. *pylori* infection alone that seems more likely associated with antral gastritis). It is demonstrated by several studies that pangastritis increases the risk of gastric neoplastic lesions [31]. These reports suggest that the coinfected subgroup (*H*. *pylori-HIV)* may be at high risk to develop gastric cancer.

We also noted a significant association between hypochlorhydria and wasting in children, with a median gastric pH of 4.5 in children with SAM. Hypochlorhydria has been widely reported in malnourished children in Bangladesh, Brazil, Nigeria, South Africa and Indonesia [32], and despite nutritional rehabilitation, this hypochlorhydria does not improve. Given the widespread nature of hypochlorhydria in developing countries as well as the observation that stimulated acid production was lower in healthy Indian men of lower socioeconomic status as compared to higher socioeconomic status [33], it has been suggested that gastric acid production and hypochlorhydria may be affected by as yet unconfirmed environmental factors in developing countries [32].

Investigations into health consequences of hypochlorhydria are ongoing. One recent example is that of the coronavirus disease 2019 (COVID-19 caused by severe acute respiratory syndrome coronavirus 2 (SARS-CoV-2). A proportion of COVID-19 patients present with gastrointestinal symptoms such as vomiting and diarrhoea, and these symptoms have been linked to late case detection and relatively more severe disease [34]. Gastrointestinal cells are known to express the angiotensin converting enzyme 2 (ACE2) receptor which facilitates SARS-CoV-2 attachment [35]. Traces of SARS-CoV-2 RNA have been found in gastrointestinal cells and although the virus may not survive at normal gastric pH levels, there is evidence that it can survive at pH levels of 3 and above, possibly leading to an increased risk of infection in individuals with hypochlorhydria [36]. Further supporting this hypothesis, there is observational evidence of a dose-dependent relationship between PPI use and COVID-19 positivity [37].

It should be noted that the adults from Study 1 had dyspepsia, and 66% of these participants reported PPI use which may have acted as a confounding factor on the proportion of patients suffering from hypochlorhydria. Hypochlorhydria maybe implicated in functional dyspepsia (FD) even in the absence of PPI therapy. A study that used 200-minute intragastric pH monitoring in patients with FD as defined by the Rome III criteria finding a small proportion of these (12.8%) had hypoacidity [38]. Hypoacidity was not observed in any other group of patients (patients with peptic ulcers, GERD or controls) and in the FDS group risk factors for hypoacidity were age>50 years (OR = 20.1) and endoscopic signs of atrophic gastritis (OR = 5.9), which is a risk factor for gastric cancer [39].

One other potential limitation of our study is the use of pH test strips to test gastric aspirate, as this only gives a measurement of gastric acidity at one point in time. Testing of gastric aspirate using pH-sensitive Litmus paper over several points in time has however been found to have excellent correlation with continuous intragastric pH probe readings [40]. Lastly, the dataset includes healthy adults and malnourished children and we, therefore, cannot comment on the population prevalence of achlorhydria or *H*. *pylori* in children.

## Conclusions

Hypochlorhydria was common amongst both children and adults in Zambia. There was a strong association between hypochlorhydria and *H*. *pylori*. The ubiquity of hypochlorhydria may be involved in gastric cancer development, as well as several other health complications including the potential impact on the bioavailability of certain antiretroviral medications which is particularly relevant in this population with high seroprevalence.
